# Supplementary material for: Cord Blood Manganese Concentrations in Relation to Birth Outcomes and Childhood Physical Growth: A Prospective Birth Cohort Study
Source: Nutrients. 2021 Nov 28;13(12):4304. doi: 10.3390/nu13124304 (PMC8705521; doi:10.3390/nu13124304)
Supplement: Supplementary file 1 [file nutrients-13-04304-s001.zip › Tab S1.pdf]

Table S1. Associations between potential covariates and prenatal Mn exposure and birth outcomes (p-values)

**Potential covariates**	Mn exposure	Birth weight	Birth length	Head circumference	PI
Maternal age	0.149	0.002	0.302	0.003	0.096
Gestational weight gain	0.313	<0.001	0.003	0.001	0.001
Gestational age	0.618	<0.001	<0.001	0.001	0.155
Pre-pregnancy BMI	<0.001	0.002	<0.001	0.036	0.359
Maternal education	0.379	0.204	0.676	0.488	0.058
Family annual income during pregnancy	0.004	0.183	0.279	0.489	0.004
Passive smoking	0.841	0.712	0.185	0.228	0.268
Vitamin use	0.117	0.223	0.725	0.876	0.049
Neonatal sex	<0.001	<0.001	<0.001	<0.001	0.524
Parity	0.234	0.008	0.564	0.017	0.044
Delivery mode	<0.001	<0.001	0.001	<0.001	0.007
Paternal BMI	0.005	0.065	0.005	0.824	0.267
Inhabitation	0.505	0.241	0.668	0.850	0.308
Anemia	0.829	0.865	0.440	0.455	0.563
	**Mn exposure**	**Weight**	**Length/Height**	**Head circumference**	**BMI**
1 year old					
Breastfeeding duration	-	0.461	0.088	0.188	0.854
2 years old					
Child's month of age	-	0.946	0.172	0.706	0.015
3 years old					
Physical activity duration	-	0.364	0.111	0.806	0.475
Child's month of age	-	0.028	0.008	0.312	0.259
6 years old					
Physical activity duration	-	0.744	0.891	0.567	0.636
Child's month of age	-	0.056	<0.001	0.067	0.634
7 years old					
Physical activity duration	-	0.356	0.987	0.150	0.299

Child's month of age	-	0.493	0.844	0.280	0.538
Total energy intake	-	0.365	0.065	0.473	0.865
8 years old					
Physical activity duration	-	0.478	0.280	0.510	0.518
Child's month of age	-	0.072	0.009	0.111	0.707

Abbreviations: Mn- manganese; PI- ponderal index; BMI- body mass index.

Pearson correlation analyses and non-parametric tests were used for comparing differences between continuous variables and categorical variables, respectively.
