# Supplementary material for: Cord Blood Manganese Concentrations in Relation to Birth Outcomes and Childhood Physical Growth: A Prospective Birth Cohort Study
Source: Nutrients. 2021 Nov 28;13(12):4304. doi: 10.3390/nu13124304 (PMC8705521; doi:10.3390/nu13124304)
Supplement: Supplementary file 1 [file nutrients-13-04304-s001.zip › Tab S2.pdf]

Table S2. Manganese concentrations (μg/L) in umbilical cord blood. (n=1179)

	GM	GSD	Min	P_{25}	P_{50}	P_{75}	P_{95}	Max	*P*
Total	29.03	1.50	6.84	22.47	29.25	37.38	54.55	316.73	
Boys	30.27	1.51	6.84	23.42	30.12	38.29	55.37	316.73	<0.001
Girls	27.71	1.47	7.64	21.49	27.93	35.38	53.80	78.42	

Abbreviations: GM: geometric mean; GSD: geometric standard deviation; Min: minimum; Max: maximum; The difference in manganese concentrations was tested by Mann-Whitney U test.
