# Supplementary material for: Cord Blood Manganese Concentrations in Relation to Birth Outcomes and Childhood Physical Growth: A Prospective Birth Cohort Study
Source: Nutrients. 2021 Nov 28;13(12):4304. doi: 10.3390/nu13124304 (PMC8705521; doi:10.3390/nu13124304)
Supplement: Supplementary file 1 [file nutrients-13-04304-s001.zip › Tab S3.pdf]

Table S3. Regression coefficients (95%CI) for associations of BMI-Z score with umbilical cord blood manganese concentrations

	Total [a]		Boys [b]		Girls [b]	
	β (95%CI)	*P*	β (95%CI)	*P*	β (95%CI)	*P*
1-year-old infants						
Ln-Mn	-0.383 (-0.668, -0.098)	0.008	-0.607 (-0.993, -0.220)	0.002	-0.144 (-0.565, 0.277)	0.504
Q_1	Ref.		Ref.		Ref.	
Q_2	0.125 (-0.195, 0.446)	0.444	0.122 (-0.316, 0.561)	0.584	0.073 (-0.397, 0.543)	0.759
Q_3	-0.131 (-0.438, 0.177)	0.405	-0.280 (-0.692, 0.132)	0.183	0.006 (-0.454, 0.466)	0.979
Q_4	-0.351 (-0.651, 0.051)	0.022	-0.486 (-0.886, -0.086)	0.017	-0.195 (-0.651, 0.260)	0.400
P-trend		0.007		0.003		0.382
2-year-old children						
Ln-Mn	-0.300 (-0.546, -0.055)	0.017	-0.283 (-0.612, 0.045)	0.091	-0.434 (-0.805, -0.062)	0.022
Q_1	Ref.		Ref.		Ref.	
Q_2	-0.007 (-0.328, 0.314)	0.966	0.336 (-0.100, 0.771)	0.131	-0.344 (-0.808, 0.120)	0.147
Q_3	-0.091 (-0.395, 0.213)	0.558	0.360 (-0.066, 0.786)	0.098	-0.572 (-0.997, -0.148)	0.008
Q_4	-0.270 (-0.559, 0.019)	0.067	-0.052 (-0.448, 0.345)	0.798	-0.517 (-0.935, -0.100)	0.015
P-trend		0.052		0.563		0.008
3-year-old children						
Ln-Mn	-0.249 (-0.477, -0.020)	0.033	-0.358 (-0.710, -0.005)	0.047	-0.166 (-0.461, 0.129)	0.269
Q_1	Ref.		Ref.		Ref.	
Q_2	0.036 (-0.238, 0.309)	0.798	0.041 (-0.379, 0.461)	0.848	-0.001 (-0.352, 0.350)	0.996
Q_3	-0.172 (-0.440, 0.096)	0.209	-0.177 (-0.587, 0.234)	0.399	-0.207 (-0.553, 0.138)	0.240
Q_4	-0.268 (-0.526, -0.009)	0.042	-0.340 (-0.734, 0.053)	0.090	-0.228 (-0.567, 0.110)	0.185
P-trend		0.016		0.044		0.112
6-year-old children						

Ln-Mn	-0.336 (-0.687, 0.015)	0.061	-0.468 (-0.967, 0.030)	0.065	-0.300 (-0.784, 0.183)	0.223
Q_1	Ref.		Ref.		Ref.	
Q_2	-0.099 (-0.473, 0.275)	0.605	-0.332 (-0.869, 0.205)	0.225	0.128 (-0.373, 0.630)	0.616
Q_3	-0.183 (-0.541, 0.175)	0.317	-0.358 (-0.856, 0.139)	0.158	-0.019 (-0.517, 0.479)	0.941
Q_4	-0.270 (-0.644, 0.103)	0.155	-0.428 (-0.948, 0.093)	0.107	-0.255 (-0.780, 0.270)	0.342
P-trend		0.138		0.117		0.326
7-year-old children						
Ln-Mn	-0.141 (-0.455, 0.173)	0.379	-0.321 (-0.796, 0.153)	0.185	0.067 (-0.333, 0.467)	0.743
Q_1	Ref.		Ref.		Ref.	
Q_2	-0.075 (-0.404, 0.254)	0.654	-0.020 (-0.498, 0.457)	0.933	-0.092 (-0.524, 0.340)	0.677
Q_3	-0.143 (-0.460, 0.174)	0.377	-0.293 (-0.750, 0.164)	0.209	0.088 (-0.337, 0.513)	0.686
Q_4	-0.245 (-0.577, 0.088)	0.150	-0.353 (-0.832, 0.127)	0.149	-0.064 (-0.508, 0.380)	0.778
P-trend		0.140		0.089		0.981
8-year-old children						
Ln-Mn	-0.128 (-0.493, 0.238)	0.493	-0.310 (-0.875, 0.254)	0.281	0.046 (-0.395, 0.488)	0.837
Q_1	Ref.		Ref.		Ref.	
Q_2	0.114 (-0.260, 0.488)	0.550	-0.201 (-0.814, 0.411)	0.519	0.348 (-0.082, 0.779)	0.112
Q_3	-0.012 (-0.378, 0.355)	0.951	-0.456 (-1.025, 0.112)	0.116	0.448 (0.001, 0.895)	0.050
Q_4	-0.139 (-0.510, 0.233)	0.465	-0.364 (-0.929, 0.201)	0.207	0.060 (-0.394, 0.513)	0.796
P-trend		0.351		0.151		0.662

[a]: Models were adjusted for maternal age at delivery, pre-pregnancy BMI, gestational age, gestational weight gain, maternal education, parity, family annual income, passive smoking, vitamin supplement during pregnancy, child's sex, child's birth weight, child's age, child's exercise, total energy intake (just for 7-year-old children).

[b]: Models were adjusted for maternal age at delivery, pre-pregnancy BMI, gestational age, gestational weight gain, maternal education, parity, family annual income, passive smoking, vitamin supplement during pregnancy, child's birth weight, child's age, child's exercise, total energy intake (just for 7-year-old children).
