# Supplementary material for: Cord Blood Manganese Concentrations in Relation to Birth Outcomes and Childhood Physical Growth: A Prospective Birth Cohort Study
Source: Nutrients. 2021 Nov 28;13(12):4304. doi: 10.3390/nu13124304 (PMC8705521; doi:10.3390/nu13124304)
Supplement: Supplementary file 1 [file nutrients-13-04304-s001.zip › Tab S4.pdf]

Table S4. Generalized estimating equation models for associations of body mass index z score with Mn exposure.

	BMI z score	
	β (95% CI)	*p*
Ln (Mn) [a]	-0.253 (-0.445, -0.060)	0.010
Q1	0	
Q2	0.038 (-0.183, 0.259)	0.733
Q3	-0.138 (-0.368, 0.093)	0.242
Q4	-0.234 (-0.460, -0.009)	0.041
p-trend		0.016
Sex-stratified analysis [b]		
Boys	-0.388 (-0.678, -0.098)	0.009
Girls	-0.120 (-0.369, 0.128)	0.343

[a]: Models were adjusted for maternal age at delivery, pre-pregnancy BMI, gestational age, gestational weight gain, maternal education, parity, family annual income, passive smoking, vitamin supplement during pregnancy, child's sex, child's birth weight.

[b]: Models were adjusted for maternal age at delivery, pre-pregnancy BMI, gestational age, gestational weight gain, maternal education, parity, family annual income, passive smoking, vitamin supplement during pregnancy, child's birth weight.
