# Supplementary material for: Cord Blood Manganese Concentrations in Relation to Birth Outcomes and Childhood Physical Growth: A Prospective Birth Cohort Study
Source: Nutrients. 2021 Nov 28;13(12):4304. doi: 10.3390/nu13124304 (PMC8705521; doi:10.3390/nu13124304)
Supplement: Supplementary file 1 [file nutrients-13-04304-s001.zip › Tab S5.pdf]

Table S5. Sensitivity analysis of Generalized estimating equation models for associations of body mass index z score with Mn exposure (Adding Ln (Pb), Ln (Cd) as covariates).

	BMI z score	
	β (95% CI)	*p*
Ln (Mn) [a]	-0.250 (-0.460, -0.040)	0.020
Q1	0	
Q2	0.018 (-0.204, 0.241)	0.872
Q3	-0.149 (-0.388, 0.090)	0.221
Q4	-0.236 (-0.476, 0.003)	0.053
p-trend		0.026
Sex-stratified analysis [b]		
Boys	-0.325 (-0.638, -0.012)	0.042
Girls	-0.159 (-0.441, 0.123)	0.270

[a]: Models were adjusted for maternal age at delivery, pre-pregnancy BMI, gestational age, gestational weight gain, maternal education, parity, family annual income, passive smoking, vitamin supplement during pregnancy, child's sex, child's birth weight, Ln (Pb), Ln (Cd).

[b]: Models were adjusted for maternal age at delivery, pre-pregnancy BMI, gestational age, gestational weight gain, maternal education, parity, family annual income, passive smoking, vitamin supplement during pregnancy, child's birth weight, Ln (Pb), Ln (Cd).
