# Supplementary material for: Cord Blood Manganese Concentrations in Relation to Birth Outcomes and Childhood Physical Growth: A Prospective Birth Cohort Study
Source: Nutrients. 2021 Nov 28;13(12):4304. doi: 10.3390/nu13124304 (PMC8705521; doi:10.3390/nu13124304)
Supplement: Supplementary file 1 [file nutrients-13-04304-s001.zip › Tab S6.pdf]

Table S6. Sensitivity analysis of Generalized estimating equation models for associations of body mass index z score with Mn exposure (Excluding low birth weight infants and preterm births).

	BMI z score	
	β (95% CI)	*p*
Ln (Mn) [a]	-0.250 (-0.443, -0.056)	0.011
Q1	0	
Q2	0.037 (-0.185, 0.260)	0.742
Q3	-0.138 (-0.369, 0.094)	0.243
Q4	-0.230 (-0.456, -0.004)	0.046
p-trend		0.018
Sex-stratified analysis [b]		
Boys	-0.384 (-0.678, -0.090)	0.010
Girls	-0.120 (-0.369, 0.129)	0.344

[a]: Models were adjusted for maternal age at delivery, pre-pregnancy BMI, gestational age, gestational weight gain, maternal education, parity, family annual income, passive smoking, vitamin supplement during pregnancy, child's sex, child's birth weight.

[b]: Models were adjusted for maternal age at delivery, pre-pregnancy BMI, gestational age, gestational weight gain, maternal education, parity, family annual income, passive smoking, vitamin supplement during pregnancy, child's birth weight.
