# Supplementary material for: Cord Blood Manganese Concentrations in Relation to Birth Outcomes and Childhood Physical Growth: A Prospective Birth Cohort Study
Source: Nutrients. 2021 Nov 28;13(12):4304. doi: 10.3390/nu13124304 (PMC8705521; doi:10.3390/nu13124304)
Supplement: Supplementary file 1 [file nutrients-13-04304-s001.zip › Tab S7.pdf]

Table S7. Generalized estimating equation models for associations of body mass index z score with Mn exposure (Including children followed up at all time points, N=83).

	BMI z score	
	β (95% CI)	*p*
Ln (Mn) [a]	-0.501 (-0.926, -0.076)	0.021
Q1	0	
Q2	0.023 (-0.512, 0.558)	0.933
Q3	0.026 (-0.436, 0.488)	0.912
Q4	-0.609 (-1.102, -0.115)	0.016
p-trend		0.014
Sex-stratified analysis [b]		
Boys	-0.822 (-1.531, -0.113)	0.023
Girls	-0.247 (-0.669, 0.174)	0.250

[a]: Models were adjusted for maternal age at delivery, pre-pregnancy BMI, gestational age, gestational weight gain, maternal education, parity, family annual income, passive smoking, vitamin supplement during pregnancy, child's sex, child's birth weight.

[b]: Models were adjusted for maternal age at delivery, pre-pregnancy BMI, gestational age, gestational weight gain, maternal education, parity, family annual income, passive smoking, vitamin supplement during pregnancy, child's birth weight.
