# Supplementary material for: Cord Blood Manganese Concentrations in Relation to Birth Outcomes and Childhood Physical Growth: A Prospective Birth Cohort Study
Source: Nutrients. 2021 Nov 28;13(12):4304. doi: 10.3390/nu13124304 (PMC8705521; doi:10.3390/nu13124304)
Supplement: Supplementary file 1 [file nutrients-13-04304-s001.zip › Tab S8.pdf]

Table S8. Comparison of manganese concentrations in cord blood in previous literature.

Reference	Location	Matrix	Year	Sample size	Median (μg/L)
The present study	China	Blood	2009-2010	1179	29.3
(Lee and Eum et al., 2021)	Bangladesh	Blood	2008-2011	1088	51.9
(Kupsco and Sanchez-Guerra et al., 2019)	Mexico	Blood	2007-2011	452	44.3
(Li and Zhuang et al., 2019)	Beijing, China	Blood	2015-2016	156	47.8
(Claus Henn and Bellinger et al., 2017)	Oklahoma, US.	Blood	2002-2011	224	43.1
(Huang and Weng et al., 2017)	Taiwan	Blood	2010-2011	145	61.68
(Arbuckle and Liang et al., 2016)	Canada	Blood	2008-2011	1419	31.8
(Guan and Wang et al., 2014)	Dalian, China	Blood	2006-2007	125	77.20
(Lin and Chen et al., 2013)	Taiwan	Blood	2004-2005	230	47.90
(Kopp and Kumbartski et al., 2012)	Bochum, Germany	Blood	2006	50	28.8
(Lin and Guo et al., 2011)	Tawain	Blood	2004-2005	1526	47.0
(Liang and Wu et al., 2019)	Ma’an Shan, China	Serum	2013-2014	3416	5.44
(Yu and Chen et al., 2016)	Shandong, China	Serum	2010-2013	377	3.4
(Yu and Zhang et al., 2014)	Shanghai, China	Serum	2008-2009	933	40

References:

Arbuckle, T. E. and C. L. Liang, et al. (2016). "Maternal and fetal exposure to cadmium, lead, manganese and mercury: The MIREC study." Chemosphere **163**: 270-282.

Claus Henn, B. and D. C. Bellinger, et al. (2017). "Maternal and Cord Blood Manganese Concentrations and Early Childhood Neurodevelopment among Residents near a Mining-Impacted Superfund Site." Environmental Health Perspectives **125** (6): 067020.

Guan, H. and M. Wang, et al. (2014). "Manganese concentrations in maternal and umbilical cord blood: related to birth size and environmental factors." European journal of public health **24** (1): 150-157.

Huang, S. H. and K. P. Weng, et al. (2017). "Maternal and umbilical cord blood levels of mercury, manganese, iron, and copper in southern Taiwan: A cross-sectional study." J Chin Med Assoc **80** (7): 442-451.

Kopp, R. S. and M. Kumbartski, et al. (2012). "Partition of metals in the maternal/fetal unit and lead-associated decreases of fetal iron and manganese: an observational biomonitoring approach." Arch Toxicol **86** (10): 1571-81.

Kupsco, A. and M. Sanchez-Guerra, et al. (2019). "Prenatal manganese and cord blood mitochondrial DNA copy number: Effect modification by maternal anemic status." Environment International **126**: 484-493.

Lee, M. and K. Eum, et al. (2021). "Umbilical Cord Blood Metal Mixtures and Birth Size in Bangladeshi Children." Environmental Health Perspectives **129** (5).

Li, A. and T. Zhuang, et al. (2019). "Heavy metals in maternal and cord blood in Beijing and their efficiency of placental transfer." Journal of Environmental Sciences **80**: 99-106.

Liang, C. M. and X. Y. Wu, et al. (2019). "Trace element profiles in pregnant women's sera and umbilical cord sera and influencing factors: Repeated measurements." Chemosphere **218**: 869-878.

Lin, C. C. and Y. C. Chen, et al. (2013). "In utero exposure to environmental lead and manganese and neurodevelopment at 2 years of age." Environ Res **123**: 52-7.

Lin, Y. Y. and Y. L. Guo, et al. (2011). "Associations between petrol-station density and manganese and lead in the cord blood of newborns living in Taiwan." Environ Res **111** (2): 260-5.

Yu, X. D. and J. Zhang, et al. (2014). "Prenatal exposure to manganese at environment relevant level and neonatal neurobehavioral development." Environ Res **133**: 232-8.

Yu, X. and L. Chen, et al. (2016). "The role of cord blood BDNF in infant cognitive impairment induced by low-level prenatal manganese exposure: LW birth cohort, China." Chemosphere **163**: 446-451.
