# Supplementary figures and images for: Cord Blood Manganese Concentrations in Relation to Birth Outcomes and Childhood Physical Growth: A Prospective Birth Cohort Study
Source: Nutrients. 2021 Nov 28;13(12):4304. doi: 10.3390/nu13124304 (PMC8705521; doi:10.3390/nu13124304)

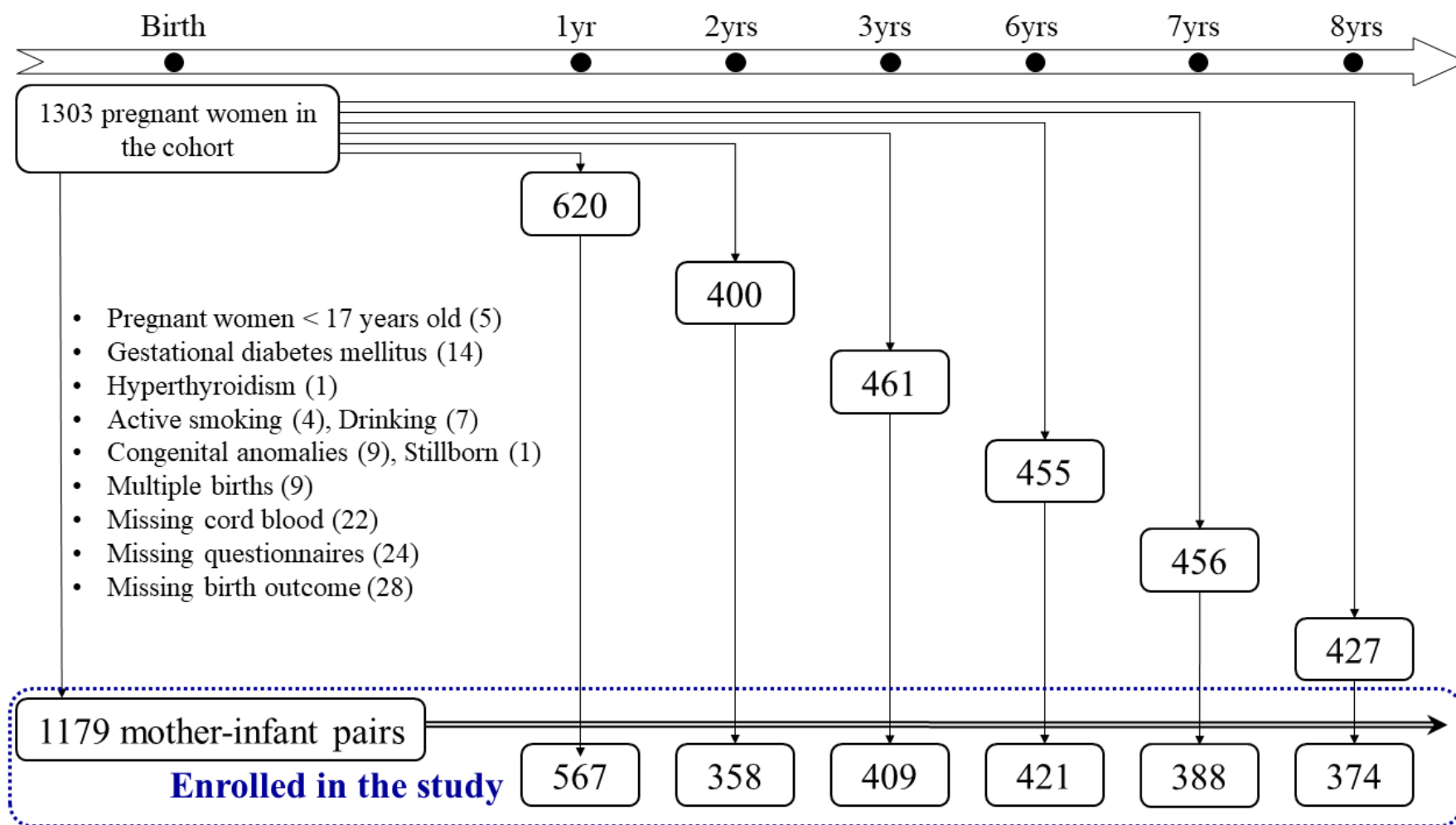

Figure S1. Recruitment and follow-up of pregnant women and their infants.

Supplement: Supplementary file 1 [file nutrients-13-04304-s001.zip › Fig S1.pdf]
